# Correction: The longitudinal impact of employment, retirement and disability status on depressive symptoms among men living with HIV in the Multicenter AIDS Cohort Study

**DOI:** 10.1371/journal.pone.0303716

**Published:** 2024-05-09

**Authors:** Deanna Ware, Sergio Rueda, Michael Plankey, Pamela J. Surkan, Chukwuemeka N. Okafor, Linda Teplin, M. Reuel Friedman

The fourth author’s name is spelled incorrectly. The correct name is: Pamela J. Surkan. The correct citation is: Ware D, Rueda S, Plankey M, Surkan PJ, Okafor CN, Teplin L, et al. (2020) The longitudinal impact of employment, retirement and disability status on depressive symptoms among men living with HIV in the Multicenter AIDS Cohort Study. PLoS ONE 15(10): e0239291. https://doi.org/10.1371/journal.pone.0239291.
