# Supplementary material for: The longitudinal impact of employment, retirement and disability status on depressive symptoms among men living with HIV in the Multicenter AIDS Cohort Study
Source: PLoS One. 2020 Oct 2;15(10):e0239291. doi: 10.1371/journal.pone.0239291 (PMC7532049; doi:10.1371/journal.pone.0239291)
Supplement: Attachment — Submitted filename: Response to Reviewers.pdf [file pone.0239291.s001.pdf]

GEORGETOWN UNIVERSITY MEDICAL CENTER

Ethan Morgan
Academic Editor
PLOS ONE
08/24/2020

Dear Ethan Morgan

Re: Submission of revised paper PONE-D-20-14823

Thank you for your email on Thursday August 6, 2020 enclosing the reviewers' comments. We have reviewed the comments carefully and revised the manuscript accordingly. Our responses are given below, with each comment in bold. Changes to the manuscript are track changed.

As corresponding author, I confirm that the formatting of the manuscript meets the requirements of the journal.

We hope the revised version is suitable for publication in *PLOS ONE*. We look forward to your decision.

Best Regards,

Deanna Ware, MPH
Corresponding Author
Georgetown University Medical Center
2115 Wisconsin Ave NW, Suite 130
Washington DC 20007
dpf38@georgetown.edu

Response to Reviewer #1:

Thank you for your review of our paper. We have answered each of your points below. Additionally, we performed missing value imputation for disability and retirement status. All models (employment, disability, and retirement) has been re-analyzed. Therefore, results vary slightly from the initial manuscript.

**Reviewer # 1 Comment**	**Author Response**
I would suggest removing retirement findings from the results as they are non-significant, or at least moving it to the end of the paragraph after the significant findings.	Thank you for your comment. I moved the non-significant result of retirement to the end of the other significant findings as suggested.
Any hypothesis as to why those 60-69 had reduced odds of depression risk?	I performed missing value imputation for retirement and disability variables. The models for employment, disability and retirement were then re-ran. Those 60-69 no longer have a statistically significant decreased odds of depression risk in all three models.
I would suggest including N's for the models in each of table 2, 3, and 4	I added the participant N and visit level N into Tables 2, 3 and 4 as suggested.

2115 Wisconsin Ave., NW | Suite 130 | Washington, DC | 20007 | 202-784-2687 | Fax: 202-784-0791

Response to Reviewer #1:

Thank you for your review of our paper. We have answered each of your points below. Additionally, we performed missing value imputation for disability and retirement status. All models (employment, disability, and retirement) has been re-analyzed. Therefore, results vary slightly from the initial manuscript.

**Reviewer # 2 Comment**	**Author Response**
The paragraph in the introduction on benefits/risks of retirement feels a bit disjointed. Revisiting this paragraph to clarify the primary point with better flow between supporting (or conflicting) studies would make it clearer why studying retirement specifically is important.	Thank you for your comment. I made changes to the paragraph introducing retirement for clarity (see lines 59-67) as suggested.
In line 75, participants are referred to as HIV-positive men. Elsewhere the preferred person-first language "People living with HIV" is used. Here, I suggest changing the language to "men living with HIV" in this sentence. People first, non-stigmatizing language should be used throughout. HIV-positive status can be changed to HIV status. HIV positive participants can be changed to participants living with HIV.	As suggested, we used person first language throughout the manuscript.
Social support is identified as a covariate but it isn't explained why this construct would be important to include in the methods or introduction but it is mentioned briefly in the discussion. I suggest making it more clear why this is included and potentially discussing it's link as an implied mechanism for linking mental health and labor engagement.	Friedman et al assessed social support in this sample of men. They found that higher social support was associated with lower levels of negative psychosocial health outcomes (which includes depression). This has been added to the manuscript to make it clear on why we chose to include it as a covariate in the models (see lines 118 to 121).
More detail about the depression measure would be helpful. How could a participant achieve a score of 16 to exceed the clinical threshold? For a	As suggested, I added the value range for the responses and the minimum and maximum scores (See lines 124-127).

reader to know this, the scoring on individual items should be reported. For example, on the CESD items are typically rated by frequency ranging from 0 to 3	
How many participants required imputation for missing employment status? Were any sensitivity checks completed to see if the imputation approach to missingness changed results? Similarly, why was this approach used for employment but not disability and retirement?	Not imputing retirement and disability was an oversight as we were initially focused on employment status. Therefore, we have imputed the missing retirement and disability values. To ensure results did not change due to imputation, we compared the model results from imputed vs non-imputed data for each of the primary outcomes: employment, retirement and disability. The estimates for the primary predictors was less than 0.001. Therefore, we can confidently state that imputing missing values did not change our results.
How was the index visit chosen and what does this mean?	Index visit is the first visit in our retroactive observation of employment, disability, retirement status and depressive symptoms.<br><br>We chose the index visit of October 1997 because it was the peak of the HAART initiation within the MACS.<br><br>Many persons living with HIV were unable to work due to disease progression. By looking at the time periods in which potent HAART was introduced, we can assess how employment trends as PLWH become healthier and possibly re-enter the workforce.
The authors make the point (without citation) that the link between labor related determinants and depression is expected to be lagged, however, it is unclear why a 2 year lag was chosen. At the aggregate level it appears justified that average sample levels of depression may lag behind major labor market shifts quite slowly but it's	D. Segel-Karpas et al used a 2-year lag on employment status and depressive symptom and decided to use a similar approach. Lines 85 to 90 explains our reasoning for the 2-year lag.

less clear why unemployment would take two years to impact mental health, particularly if in that two years employment status changes again. Because conclusions are drawn about implied causality, it is important to justify this time lag. There may be a number of confounding events or factors in that two year window that could also contribute to mental health.	"We decided to conduct time-lagged analyses to allow for the potential effects of employment, disability, and retirement on health outcomes to become salient during the observation period, and we also wanted to consider the potential adaptation to the experience of retirement or disability".
The authors refer to both within and between subjects differences in the methods, however, no within subjects analysis was conducted. The repeated measures statement in SAS typically uses subject ID to indicate within the model that some data points belong to the same participant and are thus non-independent. Repeated measures from multiple subjects do provide more robust data however it appears only between subjects results are reported in this study. Some clarification would help to make it clear that the results are between-subjects only.	We adjusted for between and within subject variances in our models. We did not report within subject differences. We will make it clear in the methods that only between subject results are reported (see line 160).
It should be stated in text that retirement was not associated with odds of depression risk. The confidence band include 1.0 and the p-value is far from the alpha = .05 significance or even p = .10 marginal significance. Likewise in the discussion it is inappropriate to draw conclusions that retirement is associated with better mental health. This is not supported by the study results.	We have made the change to reflect that retirement was not associated with the risk of depressive symptoms.
How might retirement status be confounded with age? Perhaps a different study design would be more appropriate for examining the impact of retirement. That is, younger individuals are highly unlikely to retire thus providing little variance in retirement data earlier in life. A within-person analysis would better answer the question whether an individual's mental health is different when they retire. The introduction appeared to frame the paper, in part, around aging and retirement but the methods didn't really focus on this age group or life transition. I	This is a repeated measures analysis so we adjust for with-in person variance of the predictor variables over time. That said, not all men started at the same age—so we adjusted for age for retirement (for example, to account for early vs. late retirement).

do agree with authors that understanding how retirement is related to health is important in the aging cohort of men living with HIV, but don't feel this paper really provides much insight into that process.	

2115 Wisconsin Ave., NW | Suite 130 | Washington, DC | 20007 | 202-784-2687 | Fax: 202-784-0791
